# Cytotoxicity of Bulk-Fill Composites on Stem Cells from Human Exfoliated Deciduous Teeth—An In Vitro Study

**DOI:** 10.3390/ma18163863

**Published:** 2025-08-18

**Authors:** Ralitsa Bogovska-Gigova, Nikolay Ishkitiev, Marina Miteva, Krasimir Hristov

**Affiliations:** 1 Department of Pediatric Dentistry, Faculty of Dental Medicine, Medical University of Sofia, 1431 Sofia, Bulgaria; r.bogovska@fdm.mu-sofia.bg; 2 Department of Chemistry and Biochemistry, Medical Faculty, Medical University of Sofia, 1431 Sofia, Bulgaria; nishkitiev@medfac.mu-sofia.bg (N.I.); m.miteva@medfac.mu-sofia.bg (M.M.)

**Keywords:** bulk-fill composites, stem cells, primary teeth, cytotoxicity, MTT assay, annexin V assay

## Abstract

Background: This study aimed to evaluate the cytotoxicity of bulk-fill composite materials compared to conventional compomers on stem cells from human exfoliated deciduous teeth. Methods: 90 standardized resin composite discs (4 mm thick, 4 mm diameter) were fabricated using a 3D-printed plate, comprising four bulk-fill composites (SDR, Tetric EvoCeram Bulk-Fill, VisCalor Bulk, Cention-N) and one compomer (Dyract XP). Samples were polymerized per the manufacturer’s instructions and sterilized. Stem cells were isolated from the pulp of exfoliated primary teeth. Cells were cultured and exposed to extracts of the composite materials soaked in culture medium for 24 h. Cytotoxicity was assessed using the MTT colorimetric assay, measuring cell viability via mitochondrial activity, and the Annexin V assay, quantifying apoptosis and necrosis via flow cytometry. Statistical analysis was performed using ANOVA and Tukey post hoc tests. Results: All materials significantly reduced cell viability compared to the control (*p* < 0.05), with optical density values indicating high cytotoxicity. Tetric EvoCeram exhibited the lowest necrosis and apoptosis levels, while Dyract XP showed the highest necrosis. Statistical analysis revealed no significant cytotoxicity differences among most bulk-fill composites (*p* < 0.05). Conclusion: Bulk-fill composites and conventional compomer tested exhibit comparable and significant cytotoxic effects on stem cells from human exfoliated primary teeth pulp. While these materials offer clinical advantages in pediatric dentistry due to ease and speed of application, their use underscores the dilemma of balancing operative efficiency with biological safety, and their cytotoxic profiles should be taken into consideration prior to application.

## 1. Introduction

Dental caries in childhood continues to affect a significant percentage of young children worldwide, leading to serious health consequences [1]. The anatomical and physiological features of primary teeth explain the development of carious lesions and their rapid progression in young children [2]. Primary teeth are characterized by thinner enamel and dentin layers, larger pulp chambers, and higher pulp horns that extend closer to the external tooth surface compared to permanent teeth [3]. These features result in a reduced distance between the carious lesion and the pulp, increasing the risk of pulp exposure during caries removal. The proximity of the pulp horns, particularly the mesial pulp horn, to the surface means that even moderate carious lesions can rapidly progress to pulpal involvement. Additionally, the larger pulp chamber and thinner dentinal walls make these teeth more susceptible to iatrogenic pulp exposure during operative procedures [4]. Given these anatomical and physiological considerations, the current consensus in the medical literature is to avoid nonselective (complete) carious tissue removal in primary teeth, especially in cases of deep carious lesions [5]. Restoring primary molars can be technically challenging due to their smaller size and the unique aspects of child physiology and psychology [1].

The traditional technique for using composite materials involves applying layers that are polymerized separately [6]. Each layer should have a maximum thickness of 2 mm because of the specific polymerization properties of the material [7]. The layer-by-layer technique demands precise application to avoid voids, poor bonding, or inadequate curing between layers [8]. In primary dentition, where enamel and dentin are thinner and less mineralized, achieving optimal adhesion and curing can be more difficult, potentially compromising restoration longevity [9]. Recently, there has been hope for overcoming the challenges associated with the layer-by-layer application of standard light-curing composites and compomers through the creation of bulk-fill composites. These composites, which can be light-cured or chemically cured, can be placed in layers of 4–5 mm in thickness [10].

Bulk-fill composites are available in various viscosities—low, medium, and high—and in two-phase forms [11,12]. Several studies have shown that bulk-fill composites outperform traditional composites in terms of both mechanical and biological characteristics [13,14,15,16]. Recent developments in dental material formulations have significantly improved the mechanical properties of dental composites. These advancements include enhanced toughness and wear resistance, improved adhesion to dental tissues, greater cure depth, and reduced water sorption and solubility, thereby making them suitable for demanding restorative applications [17,18].

Among the bulk-fill composites gaining popularity are thermoviscous options, such as VisCalor Bulk (VOCO, Cuxhaven, Germany). These composites are highly viscous at room temperature but become more fluid when heated to 68 °C in a composite oven or special dispenser (thermoviscous technology). VisCalor Bulk can be polymerized in layers of up to 4 mm thick with low polymerization stress. It exhibits stable mechanical properties, a low degree of water absorption and utilizes Bis-GMA and aliphatic dimethacrylate as its resin binder [19,20].

Moreover, a new powder–liquid bulk-fill composite has been developed, consisting of resin and a liquid containing alkaline fillers (alkasite) [21]. This bioactive restorative material releases significant amounts of fluoride and calcium ions at low pH levels, depositing minerals in the form of calcium phosphate and calcium fluoride [21]. These properties make it a promising option for pediatric restorations.

Bulk-fill composites offer several advantages, particularly their quick and easy application protocol [11]. The benefits of a shorter clinical procedure are even more significant in pediatric dentistry [22], as shorter treatment visits are associated with better cooperation from children [23,24]. However, if the polymerization of composite resins is incomplete, unreacted monomers may be released into the oral environment [25]. Immediately after completing composite restorations, these monomers can be detected in saliva, dentin, and even pulp, which can have adverse effects on oral structures [25,26].

Differential toxicity mechanisms in bulk-fill dental composites are primarily driven by the chemical structure and hydrophilicity of the monomers used. Hydrophilic monomers, such as TEGDMA and HEMA, tend to have higher water sorption and solubility, which facilitates their leaching from the polymerized composite into the oral environment. This increased elution correlates with greater cytotoxicity, as these monomers can penetrate biological membranes more readily and disrupt cellular function, leading to genotoxic and allergic responses [27,28].

In contrast, more hydrophobic monomers, such as BisEMA, exhibit lower water sorption and solubility, resulting in reduced elution and lower cytotoxic potential. The degree of conversion also plays a role; monomers with lower conversion rates (e.g., BisGMA) may leave more residual monomer available for elution, but hydrophilicity remains a stronger predictor of toxicity due to its impact on diffusion and bioavailability [29].

The correlation between monomer hydrophilicity and toxicity is further supported by studies showing that TEGDMA, a highly hydrophilic monomer, is consistently detected in eluates from both bulk-fill and conventional composites and is associated with pronounced cytotoxic effects on human cells [30]. Additionally, finishing and polishing procedures can reduce the quantity of residual monomer, but hydrophilic monomers are still more likely to be released even after such interventions [31].

Primary teeth contain dental pulp stem cells, specifically stem cells from human exfoliated deciduous teeth (SHED), which are multipotent and capable of differentiating into various cell types, such as odontoblasts, osteoblasts, and neural cells [32]. These cells play a critical role in tissue repair, regeneration, and potential therapeutic applications. Cytotoxic effects from unreacted monomers, such as bisphenol A-glycidyl methacrylate (Bis-GMA) or triethylene glycol dimethacrylate (TEGDMA), released from incompletely polymerized composites, can adversely affect SHED. Unreacted monomers can induce oxidative stress, disrupt cellular metabolism, and trigger apoptosis or necrosis in SHED [33]. Studies show that monomers, such as TEGDMA, can reduce cell viability by damaging mitochondrial function and increasing reactive oxygen species production, compromising stem cell survival [34].

The cytotoxicity of dental restorative materials to dental pulp cells can be assessed using a combination of in vitro, ex vivo, and advanced tissue engineering-based models [35]. Standard in vitro assays include the use of cultured human dental pulp cells or dental pulp stem cells exposed to material extracts, with cell viability measured by MTT and cell death assessed by flow cytometry (Annexin V). Additional endpoints such as cell morphology (immunofluorescence), cell attachment (flow cytometry), and oxidative stress are also commonly evaluated [36].

Given the introduction of bulk-fill composites in clinical practice for the restoration of primary teeth and their rapid and convenient handling for young patients, this study investigates the cytotoxicity of some commonly used bulk-fill materials in children. The aim of the study is to assess the cytotoxicity of bulk-fill composite materials on stem cells from human exfoliated deciduous teeth (SHEDs). The null hypothesis was that the cytotoxicity of different types of bulk-fill composite resins would not differ from each other and from that of conventional compomers used to restore primary teeth

## 2. Materials and Methods

**Figure 1:**
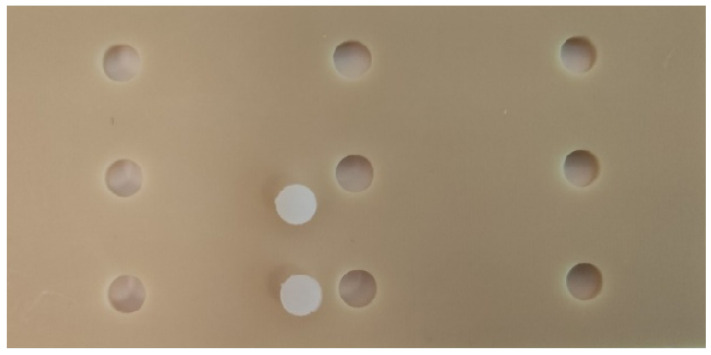
3D-printed plate and prepared flat standardized bulk-fill composite discs.

Using the 3D computer modeling program Tinkercad (Autodesk, San Rafael, CA, USA), a proprietary model of a special plate with a thickness of 4 mm and holes with a diameter of 4 mm was created (Figure 1). Ten plates were printed from it, each with nine holes (4 × 4 mm). The plate with the holes was used to prepare 90 discs—samples of composite material—18 of each material. Four bulk-fill composite restorative materials and one compomer were used in the study.

The materials used in the study are presented in Table 1.

All samples were prepared according to the manufacturer’s instructions, then applied to the holes of the 3D printed template. The composite samples were photopolymerized using a Freelight 2 Elipar™ LED curing light (3M ESPE, Athlone, Ireland) with an irradiance of 1000 mW/cm^2^ and a wavelength range of 430–480 nm, as specified by the manufacturer. Each sample was cured for 20 s per side, ensuring adequate polymerization in accordance with the manufacturers’ instructions for the respective materials. The specimens were then sterilized for 30 min per side by UV exposure.

### 2.1. Isolation of Stem Cells from Human Exfoliated Deciduous Teeth

The study used primary teeth extracted soon before the physiological time for their exfoliation. All extracted teeth were collected only after obtaining informed consent from the parents, which covered both the extraction of the teeth and their inclusion in the experiment. The study was conducted in accordance with the Declaration of Helsinki and was approved by the Ethics Committee of the Medical University of Sofia, Bulgaria (protocol No. 17/24 June 2024).

The residual pulp obtained after tooth extraction was carefully removed using dental excavators. It was then placed in a cell culture medium and transported to the laboratory. Once there, the pulp was washed three times with phosphate-buffered saline (PBS) and placed in a solution containing 3 mg/mL collagenase type I and 4 mg/mL dispase, at a volume of 0.5–1 mL, and incubated for one hour at 37 °C, with 5% CO_2_ and 50% humidity. After incubation, the suspension was centrifuged for 4 min at 3000 rpm. The supernatant, or surface liquid fraction, was carefully removed. The precipitated cells were then resuspended in 1 mL of culture medium and passed through a sterile sieve with a pore size of 70 µm (BD Falcon, Heidelberg, Germany). This process resulted in a suspension of individual cells that adhered to the bottom of the culture dish where they were seeded. The cells were seeded in 2 cm diameter plastic Petri dishes (Greiner Bio-One, Frickenhausen, Germany) with a culture medium supplemented with antibiotics and 20% fetal bovine serum at 37 °C, 5% CO_2_, and 50% humidity. Once the cells reached 80% confluence, they were trypsinized and transferred to a new dish. To do this, the existing culture medium was first aspirated, and the culture was washed with phosphate-buffered saline. A 0.05% trypsin/EDTA solution was added to the dish for 10 min. This was followed by three washes with phosphate-buffered saline to collect the detached cells. The resulting cell suspension was then centrifuged in 15-mL plastic tubes for 4 min at 3000 rpm. The precipitated cells were resuspended and seeded into a new dish at a density of 5000–10,000 cells/cm^2^, with antibiotics and 10% fetal bovine serum added to the culture medium. Cells from the third passage were used for the experiment.

### 2.2. Cytotoxicity of Bulk-Fill Resin Composite Materials Assessment

Each composite sample was immersed for 24 h in 1.2 mL of Dulbecco’s Modified Eagle Medium (DMEM) supplemented with 10% fetal bovine serum. SHEDs were seeded in a 96-well plate at a density of 10,000 cells/well in 100 µL DMEM at 37 °C, 5% CO_2_. After 24 h, the culture medium was removed and replaced with the medium in which the light-cured samples of the resin composites were stored. The cells were incubated for another 24 h. The following groups were compared:

- Group 1—cells cultured in a medium in which SDR was immersed;
- Group 2—cells cultured in a medium in which Tetric EvoCeram Bulk-Fill was immersed;
- Group 3—cells cultured in a medium in which VisCalor Bulk was immersed;
- Group 4—cells cultured in a medium in which Cention N was immersed;
- Group 5—cells cultured in a medium in which Dyract XP was immersed;
- Group 6 (Positive Control)—Cells Cultured in Dubbeco Modified Medium (DMEM) supplemented with 10% fetal bovine serum.

### 2.3. MTT Cell Proliferation Assay

The extracts were removed, and 100 µL of MTT solution (0.5 mg/mL in DMEM) was added (MTT Cell Proliferation Assay Kit, Novus Biologicals, Centennial, CO, USA). The cells were incubated for 4 h at 37 °C, allowing viable cells to reduce MTT to formazan. The MTT solution was removed, and 100 µL of DMSO was added to dissolve the formazan crystals. The plate was gently shaken for 10 min to ensure complete dissolution. The absorbance was measured at 590 nm (with a reference wavelength of 650 nm) using a microplate reader (Varioskan plate reader, Thermo Electron Corporation, Waltham, MA, USA). The background absorbance (material extract without cells) was subtracted to correct for material interference. Cell viability was calculated based on the measured absorbance, proportional to the number of viable cells.

### 2.4. Annexin V Apoptosis Assay

Annexin V staining (Annexin V Apoptosis Kit, Novus Biologicals, Centennial, CO, USA) was used to determine apoptotic and necrotic cell percentages following a published protocol [37]. To distinguish apoptotic from necrotic cells, co-staining with propidium iodide was used. Flow cytometry software (FlowJo, Becton Dickinson, v. 11, NJ, USA) quantified the percentage of cells in each category (apoptotic and necrotic). The total percentage of apoptotic and necrotic cells for each treatment condition was calculated and compared between the groups.

### 2.5. Statistical Analysis

The data followed a normal distribution (Shapiro–Wilk test, *p* > 0.05) with homogeneous variances (Levene’s test, *p* > 0.05). A one-way ANOVA was conducted to compare the optical density values and the percentages of necrotic and apoptotic cells. Tukey’s test was applied for multiple comparisons within the different groups. Statistical analyses were performed using SPSS, Version 19.0 (IBM Corp., Armonk, NY, USA). Differences among experimental groups were deemed significant at *p* < 0.05.

## 3. Results

**Figure 2:**
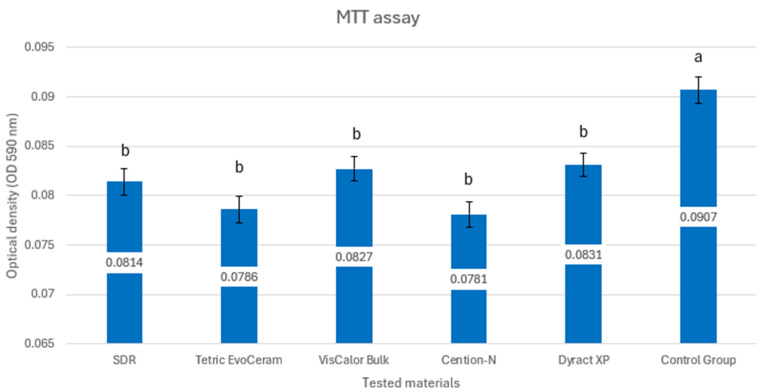
MTT assay on the cytotoxicity of bulk-fill composites. Different lowercase letters a and b indicate a significant difference.

Figure 2 presents the results of the colorimetric test with tertazolium salt.

The control group exhibited a significantly higher mean OD value compared to all test groups (*p* < 0.05). Untreated cells maintained high cell viability, while all tested dental materials induced significant cytotoxicity, as indicated by the low optical density values.

Among the test groups, Group 5 (Dyract XP) had the highest mean OD (0.0831 ± 0.0011), while Group 4 (Cention-N) had the lowest (0.0781 ± 0.0013). However, the differences in OD values among Groups 1–5 are small, suggesting comparable cytotoxicity levels across these materials.

**Figure 3:**
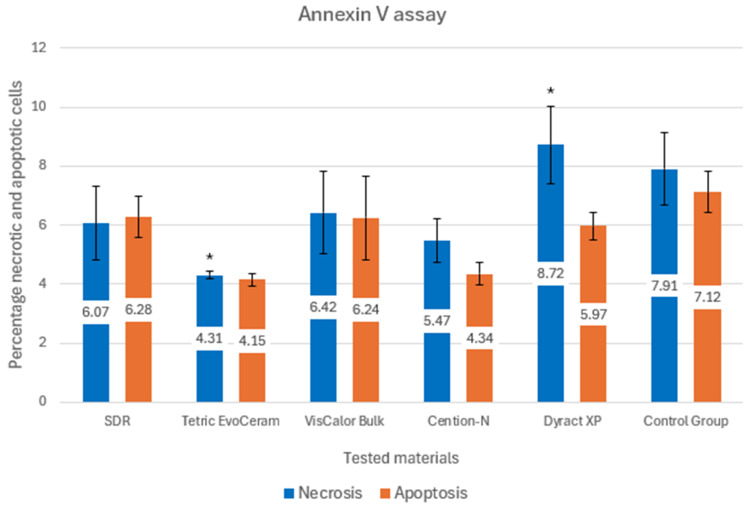
Annexin V assay for quantitation of apoptosis and necrosis. *—significant difference.

Figure 3 presents the results from the annexin V assay.

The mean percentage of necrotic cells ranged from 4.31 ± 0.12% (Group 2: Tetric EvoCeram) to 8.72 ± 1.31% (Group 5: Dyract XP) across the test groups. Group 5 (Dyract XP) exhibited the highest necrosis, while Group 2 showed the lowest.

The mean percentage of apoptotic cells ranged from 4.15 ± 0.21% (Group 2: Tetric EvoCeram) to 6.28 ± 0.68% (Group 1: SDR). Group 2 had the lowest apoptosis and Group 5 (Dyract XP) showed a relatively high apoptosis rate (5.97 ± 0.47%) among the test groups.

Most pairwise comparisons among test groups showed no significant differences (*p* > 0.05), suggesting that the necrotic effects of SDR, Tetric EvoCeram, VisCalor Bulk, and Cention-N are comparable. A significant difference was observed between Group 2 (Tetric EvoCeram) and Group 5 (Dyract XP), indicating that Dyract XP induced significantly more necrosis than Tetric EvoCeram.

## 4. Discussion

This study evaluated the cytotoxicity of four bulk-fill composite materials (SDR, Tetric EvoCeram Bulk-Fill, VisCalor Bulk, Cention-N) and one compomer (Dyract XP) on pulp cells from primary teeth, using MTT and Annexin V assays. The results show that there is no significant difference among the tested groups; thus, the null hypothesis was accepted.

It is essential to preserve the vitality of the dental pulp by using biocompatible materials, especially when restorations cover the dentin closest to the pulp [38]. Bulk-fill materials are typically applied in layers of 4 mm or more and must be light-cured at a high light intensity [39]. However, this can lead to risks of incomplete polymerization of the material, resulting in a higher concentration of unpolymerized monomers, which can have cytotoxic effects [39]. The cytotoxicity of bulk-fill composite materials is influenced by several factors, including the specific material and its chemical composition, the thickness of the applied layers, and the amount of ions released, all of which significantly impact their cytotoxic potential [25,40].

Uncured methacrylic binders in dental bulk-fill composites exert cytostatic effects primarily through the release of residual monomers such as Bis-GMA, UDMA, and TEGDMA, which can leach from incompletely polymerized material and interfere with cellular metabolism and proliferation [41,42].

The mechanism involves the diffusion of these unreacted monomers into surrounding tissues, where they disrupt mitochondrial function, induce oxidative stress, and impair cell viability, particularly in pulp and fibroblast cells [43,44].

The cytostatic action is dose-dependent and correlates with the degree of conversion: lower polymerization efficiency leads to higher residual monomer release and greater cytotoxicity [45]. Methacrylic monomers can inhibit cell proliferation by causing mitochondrial dysfunction and apoptosis, as demonstrated in vitro with fibroblast and pulp cell cultures exposed to eluates from uncured or partially cured composites [46]. TEGDMA is noted for its high cytotoxicity, while UDMA and Bis-GMA also contribute to adverse cellular effects [44].

The cytostatic effect is mitigated as the degree of conversion increases, reducing the amount of leachable monomer [47]. Bulk-fill composites are engineered for deeper curing, but incomplete polymerization at greater depths can still result in significant monomer elution and cytostatic activity [48]. Strategies to reduce cytostatic effects include optimizing photoinitiator concentration, improving monomer conversion, and using additives, such as N-acetyl-cysteine, to neutralize reactive species [42]. Overall, the cytostatic mechanism is a direct consequence of residual methacrylic monomer release from uncured or under-cured composite layers, leading to impaired cell proliferation and viability in exposed tissues [42].

The methylthiazolyldiphenyl-tetrazolium bromide (MTT) assay is a widely used colorimetric method for assessing the cytotoxicity of dental materials on various cell types. The assay measures mitochondrial metabolic activity as an indicator of cell viability, making it suitable for evaluating the effects of dental materials on cells derived from primary teeth, such as stem cells from human exfoliated deciduous teeth (SHEDs) and dental pulp stem cells (DPSCs) [49,50,51]. Studies have demonstrated that the MTT assay is effective for detecting cytotoxic effects of bulk fill composites on human dental pulp stem cells, with results showing material- and time-dependent reductions in cell viability after exposure to composite eluates [52]. The MTT assay is sensitive, reproducible, and allows for rapid screening of multiple samples, but results can be influenced by factors such as material composition, extract preparation, and assay conditions.

The MTT assay results from our study showed that all tested materials significantly reduced cell viability compared to the control group, with optical density values ranging from 0.078 to 0.083, compared to 0.0907 for the control (Figure 2). This indicates high cytotoxicity across all materials, as the low OD values reflect a substantial decrease in metabolic activity. Most test groups showed no significant differences in cytotoxicity (*p* > 0.05), supporting the null hypothesis.

Bulk-fill composite materials are widely used in dental restorations due to their ability to be placed in thicker increments and cured efficiently. However, their cytotoxicity has been a subject of investigation. Studies have shown that bulk-fill composites exhibit dose-dependent cytotoxicity. For instance, Junqueira et al. found that extracts from Filtek™ One Bulk Fill, Tetric Evoceram^®^ Bulk Fill, and Coltene Fill-Up! resins were cytotoxic at concentrations above 1%, with Coltene Fill-Up! being the most cytotoxic [53]. Similarly, Haugen et al. reported that conditioned media from Filtek™ Bulk Fill Flowable Restorative and Tetric EvoCeram were cytotoxic to primary human osteoblasts [30]. The cytotoxicity of these materials is influenced by several factors, including the chemical composition, thickness of the composite, and the degree of polymerization. Toh et al. demonstrated that thicker specimens (4 mm) of bulk-fill composites had higher cell viability compared to standard composites, but not all bulk-fill RBCs showed high cell viability at this thickness [54]. Oxidative stress and depletion of cellular glutathione (GSH) are suggested mechanisms for the cytotoxicity observed in resin-based composites [55]. Additionally, Lee et al. highlighted that the depth of cure affects cytotoxicity, with deeper layers (4–6 mm) showing more cytotoxic effects [56].

The Annexin V assay is a widely used method to evaluate cytotoxicity by detecting early apoptosis and necrosis in cells by binding to phosphatidylserine exposed on the outer leaflet of the plasma membrane [35]. The medical literature demonstrates that annexin V-based assays are a standard method for evaluating apoptosis in cytotoxicity studies of dental materials, including bulk-fill composites. For example, in studies of bioactive dental materials relevant to pulp capping, flow cytometry with FITC-labeled annexin V has been used to quantify apoptotic cell populations after exposure to composite eluates. This approach allows for sensitive detection of early apoptotic events, which is critical for understanding the biocompatibility of materials placed in close proximity to the dental pulp [35].

Specifically, when human dental pulp cells or stem cells are exposed to bulk-fill composite extracts, annexin V assays can reveal increased apoptosis, particularly with materials that exhibit higher cytotoxicity. The degree of annexin V positivity correlates with the extent of cellular stress and apoptotic induction, providing mechanistic insight into the mode of cell death (apoptosis vs. necrosis) induced by leachable components from these composites [35].

The Annexin V assay, which distinguishes between necrosis and apoptosis, revealed that all materials induced both types of cell death (Figure 3). Tetric EvoCeram Bulk-Fill exhibited the lowest levels (4.31% necrosis, 4.15% apoptosis), while Dyract XP showed the highest necrosis (8.72%). Statistical analysis of the Annexin V data indicated no significant differences among most test groups, except for a significant difference in necrosis between Tetric EvoCeram and Dyract XP (*p* = 0.031). This suggests that while the materials generally have similar cytotoxic effects, Dyract XP may cause more direct cell membrane damage.

Bulk-fill composites often contain different monomers and fillers compared to traditional composites. For example, bulk-fill materials, such as SDR Flow release Bisphenol A-glycidyl methacrylate (BisGMA) and other monomers, which can contribute to cytotoxicity. Traditional composites, such as Tetric EvoCeram (TEC) also release similar monomers, but the specific composition can vary, affecting cytotoxicity levels [30]. Bulk-fill composites are designed to be placed in thicker increments (up to 4–6 mm) and cured efficiently. Studies have shown that thicker specimens of bulk-fill composites (4 mm) generally exhibit higher cell viability compared to traditional composites at similar thicknesses. However, not all bulk-fill materials maintain high cell viability at these thicknesses, indicating variability among products [54,55].

The degree of polymerization significantly affects the cytotoxicity of bulk-fill composite materials compared to traditional composites. Higher degrees of polymerization generally result in lower cytotoxicity due to reduced release of unreacted monomers, which are cytotoxic. Bulk-fill composites are designed to achieve a higher degree of polymerization even at greater depths (up to 4–6 mm) compared to traditional composites. Studies have shown that bulk-fill composites, such as SDR Flow and Filtek™ Bulk Fill, exhibit higher degrees of conversion and depth of cure compared to traditional composites, such as Tetric EvoCeram (TEC) [30,56,57]. This higher degree of polymerization in bulk-fill composites leads to a lower release of cytotoxic monomers, thereby reducing their cytotoxicity. For instance, Haugen et al. demonstrated that the degree of conversion and depth of cure were highest for SDR, followed by FBF, and then TEC. Despite this, conditioned media from FBF and TEC were cytotoxic to primary human osteoblasts, indicating that the degree of polymerization plays a crucial role in cytotoxicity [30]. Similarly, Zorzin et al. found that bulk-fill composites achieved sufficient polymerization properties at 4 mm depth, which is critical for reducing cytotoxic effects [57].

To improve the degree of curing in bulk-fill dental composites, applying these materials in thinner layers (e.g., 2–3 mm increments) is recommended, particularly for deeper restorations. This approach enhances light penetration through the composite, facilitating a higher degree of monomer conversion and reducing the amount of residual unpolymerized monomer, which can contribute to cytotoxicity and compromised mechanical properties [58]. Incremental layering allows for better control of polymerization shrinkage stress and ensures more uniform curing throughout the restoration. Techniques such as the use of high-intensity light-curing units with extended curing times (e.g., 20–40 s per layer) can further optimize the degree of conversion in thinner layers [59]. Additionally, employing a soft-start or pulse-delay curing protocol may minimize shrinkage stress while maintaining adequate curing depth, particularly for composites containing hydrophilic monomers with higher polymerization rates [60]. These strategies can be practically implemented in clinical settings to improve the longevity and biocompatibility of bulk-fill composite restorations.

The high cytotoxicity observed in all test groups is concerning, especially for pediatric dentistry, where pulp cells are more sensitive. The use of primary tooth pulp stem cells is relevant for pediatric applications, but the in vitro setup—exposing cells to material extracts—may not fully mimic clinical conditions, where dentin and saliva could influence outcomes.

While the in vitro cytotoxicity observed in this study indicates a potential for cellular damage, the clinical environment introduces factors that may modulate these effects. The presence of dentin barriers, salivary buffering, and pulpal blood flow can reduce the direct exposure of pulp tissue to leachable components from restorative materials [61,62]. In pediatric patients, the pulp is particularly sensitive due to its higher cellularity and thinner dentin layer, which may amplify the impact of cytotoxic effects in deep restorations [63]. Our findings underscore the importance of cautious material selection in pediatric dentistry, prioritizing materials with lower cytotoxic potential, especially for procedures involving close proximity to the pulp. Clinicians should consider the balance between material performance and biocompatibility to ensure the long-term vitality of primary teeth.

The comparable cytotoxicity profiles among the tested materials suggest that no single material is significantly less toxic, though Group 5 (Dyract XP) exhibited slightly higher necrosis rates, particularly when compared to Group 2 (Tetric EvoCeram). This difference may be clinically relevant in procedures involving larger or deeper restorations, where pulp exposure to cytotoxic components is more likely. To minimize potential risks, clinicians are advised to employ protective strategies, such as using incremental layering techniques or applying liners (e.g., calcium hydroxide or glass ionomer) to act as a barrier between the restorative material and the pulp. These approaches can reduce direct contact and mitigate cytotoxic effects, particularly in young patients, where preserving pulp vitality is critical for tooth development and longevity.

### Limitations

While this in vitro study provides valuable insights into the cytotoxicity of bulk-fill composites (SDR, Tetric EvoCeram Bulk-Fill, VisCalor Bulk, Cention-N) and a conventional compomer (Dyract XP) on stem cells from exfoliated human deciduous teeth, several limitations should be mentioned. In vitro cytotoxicity assays, such as the MTT and Annexin V assays used in this study, evaluate direct cellular responses to material extracts under controlled conditions, which do not fully replicate the complex biological environment of the oral cavity. In vivo, the dentin barrier plays a critical role in modulating the bioavailability of leachable components, such as residual monomers or additives, by acting as a physical barrier that reduces the diffusion of these substances toward the pulp. Additionally, tissue fluid flow and salivary buffering in the oral environment can dilute or neutralize potentially toxic compounds, further mitigating their effects. The pulp’s defense and repair mechanisms, including inflammatory responses and dentinogenic activity, may also counteract or repair cellular damage caused by these materials, particularly in the dynamic setting of young pulp tissue in pediatric patients. Moreover, in vitro studies typically assess acute cytotoxicity using short-term exposure to material extracts, which may not account for the gradual release of monomers over time in vivo, potentially leading to different toxicological profiles. These factors pose challenges for directly extrapolating in vitro findings to clinical scenarios, as the cytotoxic effects observed in this study may be less pronounced or altered in the presence of these biological and physiological variables. Therefore, while our results highlight the potential cytotoxicity of the tested materials, further in vivo studies and long-term clinical evaluations are essential to fully understand their safety and biocompatibility in pediatric dentistry.

## 5. Conclusions

The significant cytotoxicity observed in all test groups compared to the control suggests that the dental materials tested (SDR, Tetric EvoCeram Bulk-Fill, VisCalor Bulk, Cention-N, Dyract XP) release components that adversely affect cell viability in this assay. This could be due to residual monomers, additives, or other bioactive compounds leaching from these materials.

While the bulk-fill composites exhibited comparable cytotoxicity to each other, necrosis induction was significantly lower with Tetric EvoCeram Bulk-Fill compared to the Dyract XP compomer. The lack of significant differences among most test groups indicates that these materials generally have comparable cytotoxic profiles, which is relevant for clinical decision making when selecting restorative materials.

## Figures and Tables

**Table 1:** Composition and brand name of the bulk-fill materials used to assess their cytotoxicity to SHEDs.

Samples	Manufacturer	Material	Composition
18	Dentsply Sirona, Konstanz, Germany	SDR, Low-viscosity bulk-fill resin composite	Modified UDMA, TEGDMA, dimethacrylate and trimethacrylate resin, Silanated bariumaluminofluoroborosilicate glass, silanated strontium aluminofluoro-silicate glass, surface treated fume silicas, ytterbium fluoride, synthetic inorganic iron oxide pigments, and titanium dioxide
18	Ivoclar Vivadent, Schaan, Liechtenstein	Tetric EvoCeram Bulk-Fill, High-viscosity bulk-fill resin composite	Bisphenol-Aglycidylmethacrylat (Bis-GMA), Bis-EMA, and barium glass filler
18	VOCO, Cuxhaven, Germany	VisCalor Bulk, Thermoviscous bulk-fill resin composite	Bis-GMA, aliphatic dimethacrylate, and inorganic filler
18	Ivoclar Vivadent, Schaan, Liechtenstein	Cention-N, Moderateviscosity alkasite material	Calcium-fluoro-silicate glass, barium-aluminosilicate glass, ytterbium trifluoride, copper salt and thiocarbamide-self cure initiator (Ivocerin), acyl phosphine oxidephotoinitiator, pigment, urethane dimethacrylate (UDMA), tetramethyl Xylylendiurethane dimethacrylate, Tricyclodecandimethanol dimethacrylate (DCP), polyethylene glycol 400 dimethacrylate (PEG400 DMA), initiator (hydroperoxide—selfcuring), and stabilizer
18	Dentsply Sirona, Konstanz, Germany	Dyract XP, High-viscosity compomer material	UDMA, carboxylic acid modified dimethacrylate, TEGDMA, trimethacrylate resin (TMPTMA), dimethacrylate resins, camphorquinone, ethyl-4 (dimethylamino) benzoate, butylated hydroxy toluene (BHT), strontium-aluminosodium-fluoro phosphorsilicate glass, highly dispersed silicon dioxide, strontium fluoride, iron oxide pigments, and titanium oxide pigments

## Data Availability

The original contributions presented in this study are included in this article, and further inquiries can be directed to the corresponding author.

## Abbreviations

The following abbreviations are used in this manuscript:

SHED	Stem cells from human exfoliated deciduous teeth
